# Real-time factory smoke detection based on two-stage relation-guided algorithm

**DOI:** 10.1038/s41598-022-05523-1

**Published:** 2022-02-02

**Authors:** Zhenyu Wang, Duokun Yin, Senrong Ji

**Affiliations:** School of Control and Computer Engineering, North China Electric Power University, Beijing, 102206 China

**Keywords:** Environmental impact, Electrical and electronic engineering

## Abstract

Recently, air quality analysis based on image sensing devices has attracted much attention. Since most smoke images in real scenes have challenging variances, which is difficult for existing object detection methods. To keep real-time factory smoke under efficient and universal social supervision, this paper proposes a mobile-platform-running efficient smoke detection algorithm based on image analysis techniques. We introduce the two-stage smoke detection (TSSD) algorithm based on the lightweight detection framework, in which the prior knowledge and contextual information are modeled into the relation-guided module to reduce the smoke search space, which can therefore significantly improve the performance of the single-stage method. Experimental results show that the proposed TSSD algorithm can robustly improve the detection accuracy of the single-stage method and the model has good compatibility for image resolution inputs. Compared with various state-of-the-art detection methods, the accuracy $AP_{mean}$ of our proposed TSSD model reaches 59.24$\%$, even surpassing the current detection model Faster RCNN. In addition, the detection speed of our proposed model can reach 50 ms (20 FPS), meeting the real-time requirements. This knowledge-based system has the advantages of high stability, high accuracy, fast detection speed. It can be widely used in some scenes with smoke detection requirements, such as on the mobile terminal carrier, providing great potential for practical environmental applications.

## Introduction

Air pollution source monitoring is of great importance in clean production. With the improvement of industrialization, the factory smoke pollution has become an unavoidable problem. Real-timely and accurately monitoring factory smoke is of great importance for human health and sustainable development. At present, environmental protection departments usually use online continuous emission monitoring system (CEMS)^1^ to measure the concentrations of some gases (such as sulfur dioxide, nitrogen oxides) and solid particles in smoke online, then monitor the status of factory smoke pollution emissions. However, such specific monitoring results are not accessible to common people. At the same time, CEMS severely relies on physicochemical sensors to analyze collected component by some methods, which is not always reliable.

To address above problem and achieve efficient smoke detection, we make use of the computer vision methods and propose an efficient algorithm which can locate and identify factory smoke real-timely and accurately by pictures taken by mobile phones. Note that the smoke in these pictures may not be all harmful, but the black, yellow, red, white smoke with a pungent smell usually causes severe air pollution. Through further fine-grained image recognition based on the detected smoke by our algorithm, people should be able to know whether the smoke is polluted or not. In this way, it is convenient to report the surrounding factory smoke pollution phenomenon, which has a great social supervision significance. Meanwhile, our image analysis solution can act as a supplement to the CEMS monitoring to provide a promising auxiliary means on pollution prevention and control.

For the detection of smoke in image, current research methods are mainly proposed in fire disaster warning and military fields. Traditional methods mostly used the hand-crafted features^2^ to realize image recognition through different classifiers. Their designs are complex and the detection accuracy still need to be improved. Since AlexNet^3^ won the first prize in the ImageNet Competition in 2012, deep learning method has been widely applied in image and computer vision. The newest smoke detection method based on deep learning^4^ used the effective convolutional neural network to extract image features automatically and achieved better detection accuracy. However, few works are proposed for high-accuracy location of smoke in the field of air pollution source monitoring, especially in the aspect of factory smoke pollution detection. At the same time, bad weather can also affect the performance of model. Some work related to image inpainting may solve the problem. Chen et al.^5^ provides a decent method to implement the image inpainting, which can make detection models suitable for all weather circumstances. Chen et al.^6^ also gives a useful image inpainting algorithm, using known information to restore the noisy images.

Recently, the object detection methods^7,8^ based on deep learning provide much help for real-time detection of smoke in images. The existing lightweight detection frameworks can be used to directly meet the requirements. Although they can obtain certain accuracy in most cases, the smoke detection task usually has a large scene, and existing methods may cause problems such as missed detection and false detection. In addition, some aspects are also not fully considered: (1) The shape of factory smoke is often variable and it’s easily affected by random factors such as wind, which brings certain challenges to detection; (2) The existing detection models fail to make full use of prior knowledge and ignore the inseparable relations between smoke and other objects, while these contextual relations are exactly important; (3) There may be some regions in the background that are similar to the smoke shape, which can have a bad influence for robust and accurate detection.

In order to solve the problems above, this paper proposes a two-stage relation-guided smoke detection (TSSD) algorithm. It makes full use of the contextual relation between chimney and smoke to minimize reduces of the searching space of smoke region, thereby improving the accuracy of smoke detection compared with the baseline model under the premise of ensuring real-time performance.

For this paper, the main contributions of this paper are as follows:

1. Aiming at a much more accessible industrial smoke pollution localization, this paper proposes a two-stage relation-guided smoke detection algorithm to reduce smoke searching space and improve detection accuracy of the baseline one-stage model. It could make full use of the prior knowledge and contextual relations for accurate detection. It uses pictures instead of specific sensors, making the results more accessible to people.
2. The proposed method achieves better trade-off between accuracy and speed, which can meet the requirements of real-time factory smoke detection. Extensive experimental evaluations show that our method is effective and outperforms the compared popular object detection models.
3. This paper designs a specific factory smoke image dataset with 960 high-quality images for analysis and evaluations. It provides a mobile-platform-running auxiliary method for the environmental protection, having a great social supervision significance.

The remainder of this paper is organized as follows. The related works on factory smoke detection are introduced in “Related works”. “Baseline model for smoke detection” describes the baseline model of smoke detection. In the following section, the proposed TSSD algorithm is presented in detail. The experiment and the result analysis are carried out strictly in “Experiments”. The conclusion is formed in the last section.

## Related works

The section introduces the related works in succession, including the visual based smoke detection and some object detection methods.

### Visual based smoke detection

The research on smoke detection using image can be divided into two categories: traditional methods based on hand-designed features, deep learning methods using neural network to extract features.

For traditional methods, hand-designed features were usually as input into some machine learning methods such as the Support Vector Machine^9,10^, the shallow Neural Network^11^, and AdaBoost^12^. However, the smoke features are complex and burdensome to obtain by cascade steps. Deep learning methods can automatically extract these features and effectively achieve higher accuracy. Yin et al.^13^ proposed the deep normalized convolutional neural network, embedding the normalized layer into convolutional network to realize better smoke detection. Yin et al.^14^ applied recurrent neural networks to this task, which effectively captured the contextual information of smoke long-range movement. Gu et al.^15^ developed a deep dual-channel neural network and achieved better smoke detection by the concatenation of the sub-network of extracting different-level features. However, these methods didn’t aim at the factory smoke detection task and also failed to achieve the high-accuracy location of the smoke object. Therefore, we investigated the general mainstream object detection frameworks.

### Object detection methods

The popular object detection frameworks based on deep learning can be roughly divided into two categories: proposal-based and proposal-free methods. The proposal-based methods first generate proposal regions in the first stage, then regress and classify these regions in the second stage. The proposal-free methods directly generate the object classification information and location coordinates without proposal regions.

In terms of the two-stage detection, Girshick et al.^7^ were the earliest to propose the RCNN model for object detection. Fast RCNN^16^ was further proposed to solve some disadvantages of RCNN. To achieve end-to-end better detection, Faster RCNN^17^ was developed to achieve the integration of the feature extraction, the proposal region generation, the bounding box regression and classification. About the one-stage detection, Redmon et al.^8^ designed YOLO to directly predict the object location and category by once network inference to the original input images. SSD^18^ was developed by referring to YOLO and the different-scale idea. Based on of YOLO-V2^19^, Redmon et al.^20^ further proposed YOLO-V3 to achieve better detection performance. Recently, Bochkovskiy et al.^21^ combined different strategies to develop more complex yet accurate model YOLO-V4. Transformer is also promising in object detection field. In recent months, there has been many transformer-based object detection algorithms. Carion et al.^22^ used transformer to implement object detection first, significantly outperforming competitive baselines; Zhu et al.^23^ optimized the structure of DETR by using a small set of key sampling points, achieving better performance especially on small objects; based on DETR, Meng et al.^24^ put forward Conditional DETR, making training convergence faster. In these mainstream detection frameworks, we choose YOLO-V3 as the benchmark model of smoke detection due to its mature application in industry and good trade-off between speed and accuracy.

### Object detection with contextual relation

In the above sub-section, those detection frameworks only rely on the per-class inherent features and fail to fully introduce the contextual relevance around objects. Some works that model the contextual information into detection framework have been gradually carried out. Shrivastava et al.^25^ integrated contextual segmentation into Faster RCNN. Bell et al.^26^ embedded ION structure into Fast RCNN to capture contextual information of ROI region. Leng et al.^27^ proposed context learning network into Faster RCNN. These methods improved the detection accuracy of respective benchmark networks. In addition, some researches on explicitly modeling contextual relation between objects have also been promoted. Hu et al.^28^ proposed an object relation module to describe the relative location relation between different objects. Xu et al.^29^ used the spatial-aware graph relation network to model important semantic and spatial relation between objects. Kim et al.^30^ developed a spatial relation reasoning framework to encode object features. Chen et al.^31^ use the semantic relation network and spatial relation network to model both global semantic relation and local spatial relation respectively. These methods were separately introduced into Faster RCNN and improved its detection accuracy. Inspired by them, this paper decides to develop a detection framework merging the smoke surroundings and its contextual relation.

## Baseline model for smoke detection

This paper selects the widely adopted YOLO-V3 as the baseline model for smoke detection. The default model input size is $416\times 416$ and output has three-scale detections with the binary smoke class. For all the output boxes, non-maximum suppression (NMS) is adopted to obtain final detections. Its full procedure is listed in Algorithm 1.

### Network structure of the baseline

**Figure 1:**
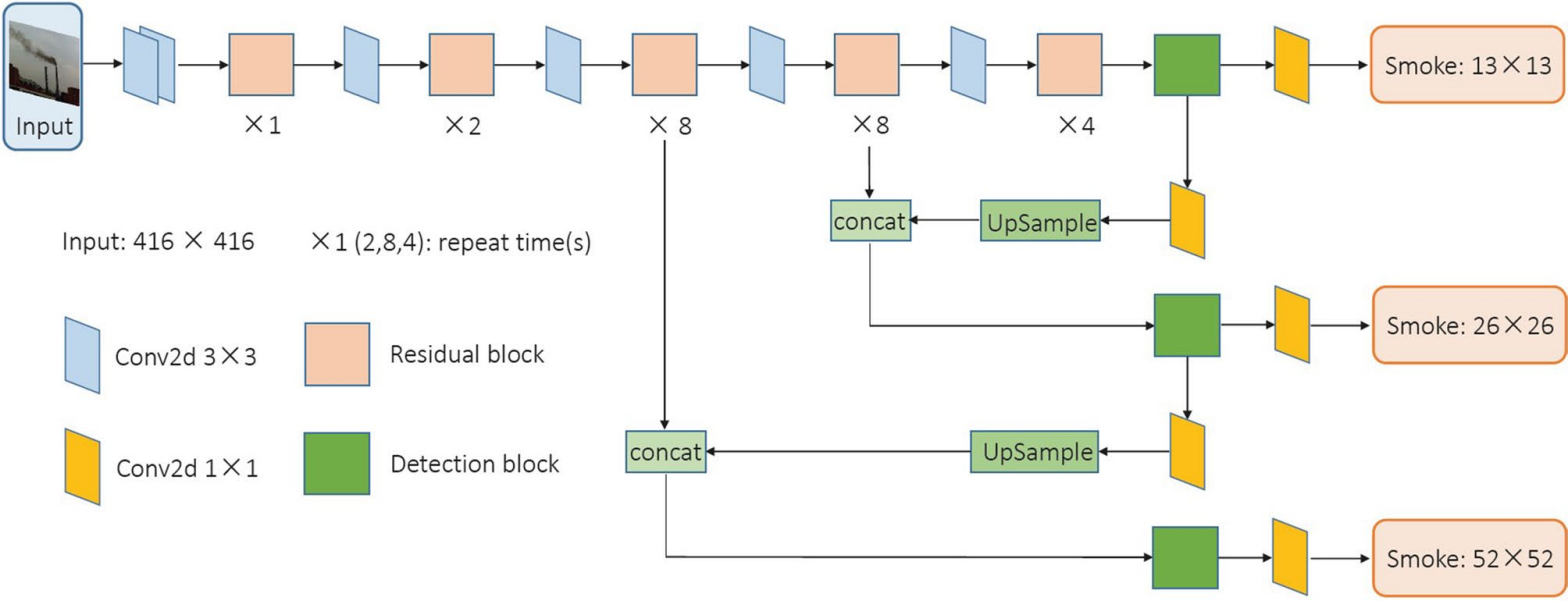
Network structure of the baseline model. It’s based on YOLO-V3 backbone. The network input is the $416\times 416$ smoke image and output has three-scale detections with the binary smoke class.

**Figure 2:**
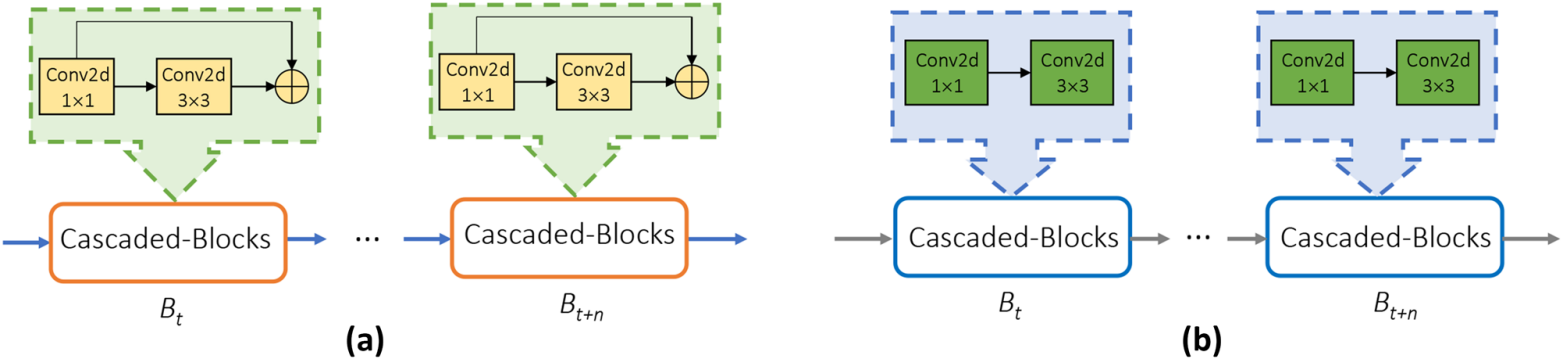
Cascaded-Blocks of Conv2d $1\times 1$ and Conv2d $3\times 3$. **(a)** Denotes ‘Residual Block‘ to extract relative low-level smoke features, where n is 1,2, 8,4, from left to right. **(b)** Denotes ‘Detection Block‘ to extract relative high-level smoke features, where n is 3. Compared with **(b)**, the residual connection of **(a)** can help to capture more feature information.

Network structure of the baseline model is shown in Fig. 1. There is a brief introduction to some of parameters and modules in this model:

’Conv2d’ means 2-dimension convolutional network; ’Residual Block’ denotes the residual-connected block of Conv2d $1\times 1$ and Conv2d $3\times 3$, as shown in Fig. 2a; ’Detection Block’ is the combined block of Conv2d $1\times 1$ and Conv2d $3\times 3$, as shown in Fig. 2b.

The feature extraction network Darknet-53 is composed of alternating Conv2d layer and Residual Block, with a 53-layer fully convolutional network structure. In Darknet-53, the convolution with step size of 2 is to replace down-sampling pooling. And the channel number of the feature map is doubled through per convolution calculation to get more abstract feature information; residual connection is introduced to mine deeper network information for better image recognition.

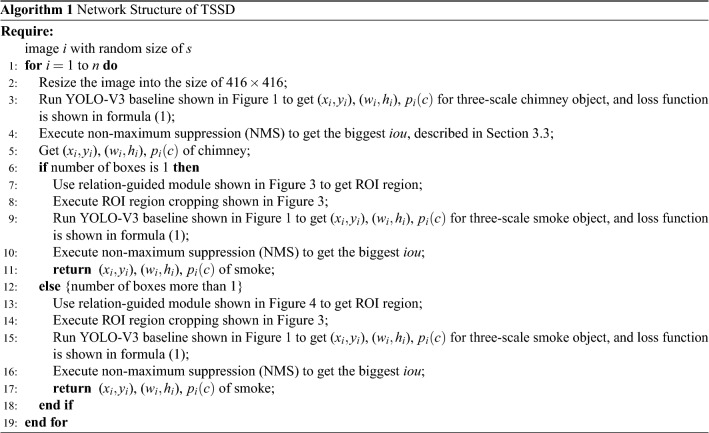



This model learns from the multi-scale fusion strategy of FPN^32^ and uses three-scale branches to detect large, medium and small objects. And the size of the output feature layer is $52\times 52$, $26\times 26$ and $13\times 13$, respectively. Detection Block has two outputs after processing these three feature layers. One is followed by Conv2d $1\times 1$ to give this-scale prediction results, and the other is up-sampled to concatenate with the previous feature layer correspondingly. The neural network can learn deep and shallow feature information to reach better feature representation of images.

### Objective function

Objective function of the baseline model is the weighted sum of bounding boxes’ coordinate loss, confidence loss, and classification loss. The whole loss calculation formula is as follows.

1 $$\begin{aligned} \begin{aligned} Loss =&\sum \limits _{i=0}^{{{s}^{2}}}{\sum \limits _{j=0}^{B}{1_{ij}^{obj}\times }}(2-{{w}_{i}}\times {{h}_{i}})\times [(1-giou[({{x}_{i}},{{y}_{i}},{{w}_{i}},{{h}_{i}}),({{{\hat{x}}}_{i}},{{{\hat{y}}}_{i}},{{{\hat{w}}}_{i}},{{{\hat{h}}}_{i}})]] \\&- \sum \limits _{i=0}^{{{s}^{2}}}{\sum \limits _{j=0}^{B}{1_{ij}^{obj}\times \alpha |1-{{C}_{i}}^{j}{{|}^{\gamma }}}}\times [{{{\hat{C}}}_{i}}^{j}log({{C}_{i}}^{j})+(1-{{{\hat{C}}}_{i}}^{j})log(1-{{C}_{i}}^{j})] \\&- \sum \limits _{i=0}^{{{s}^{2}}}{\sum \limits _{j=0}^{B}{1_{ij}^{noobj}\times (1-\alpha )|0-{{C}_{i}}^{j}{{|}^{\gamma }}\times [{{{\hat{C}}}_{i}}log({{C}_{i}})}}+(1-{{{\hat{C}}}_{i}})log(1-{{C}_{i}})] \\&- \sum \limits _{i=0}^{{{s}^{2}}}{1_{ij}^{obj}\sum \limits _{c\in class}{[{{{\hat{p}}}_{i}}(c)log({{p}_{i}}(c))+(1-{{{\hat{p}}}_{i}}(c))log(1-{{p}_{i}}(c))]}} \end{aligned} \end{aligned}$$

where the first item is bounding boxes’ coordinate loss, and *giou*^33^ method is introduced to measure the location bias between predicted and true boxes. The sum of the third and fourth items is the confidence loss and focal loss^34^ is introduced to measure this bias. The fifth item is the classification loss of bounding boxes containing objects.

Here, we give a brief introduction to parameters in the formula:

*s* denotes the grid size of the final feature map, so here ${{s}^{2}}$ corresponds to $13\times 13$, $26\times 26$ and $52\times 52$; *B* is the number of predicted bounding boxes generated by each cell grid; $1_{ij}^{obj}$ ($1_{ij}^{noobj}$) indicates the jth bounding box in the ith grid is (not) to detect this object; (*x*, *y*) denotes center coordinates of the bounding box; (*w*, *h*) denotes its width and height; *C* is the number of classification; $C_{i}^{j}$ is an indicator of the jth bounding box’s confidence score in the ith grid; *p* is the predicted probability of different categories; $\alpha$ is the balance parameter of positive and negative samples; $\gamma$ is the weight coefficient of samples difficult and easy to classify; $\alpha$ and $\gamma$ are manually empirical values.

### Post processing

To reduce redundant prediction boxes generated by the networks, we adopt post processing of non-maximum suppression (NMS)^35^. In the detection process, the baseline model will generate multiple bounding boxes for the same target. The aim of NMS is to keep one-and-only bounding box with the largest confidence as the final detection result of the target. Among all predicted boxes from the baseline model, we first sort them by the classification probability and keep the box with the highest score. Then we calculate the IoU between it and other boxes to discard those boxes whose IoU is larger than the set threshold value. We repeat this process in the remaining boxes and keep the highest-score box as output each time. Finally, these highest-score boxes come to be predicted outputs. In NMS, IoU denotes the overlap ratio between ground truth box A and prediction box B. We denote their overlap area as ${A\cap B}$ and total combined area as ${A\cup B}$. Its calculation formula is:

2 $$\begin{aligned} IoU = \frac{A\cap B}{A\cup B} \end{aligned}$$

## Two-stage smoke detection (TSSD) framework

To improve the detection accuracy of the baseline model, this paper proposes a two-stage smoke detection (TSSD) algorithm. The relation-guided module is the core of this algorithm.

### Overview of TSSD

Inspired by the attention mechanism and contextual awareness, this paper proposes to detect the stable chimney object firstly then to detect smoke in the region above the chimney location, which can improve the accuracy of the neural network regression. This strategy can greatly reduce the influence of disruptive factors such as wind, rain and other weather conditions. In the first stage, a YOLO-V3 based detector predicts the chimney location in the image. In the second stage, the designed relation-guided module analyzes these prediction results to reduce smoke searching region, then the detector locates the smoke object in this region and maps the predicted location back to the original image as the final outputs. As a supplement, multi-heads predictions exist in the detector and they are concatenated to be processed with non-maximum suppression.

**Figure 3:**
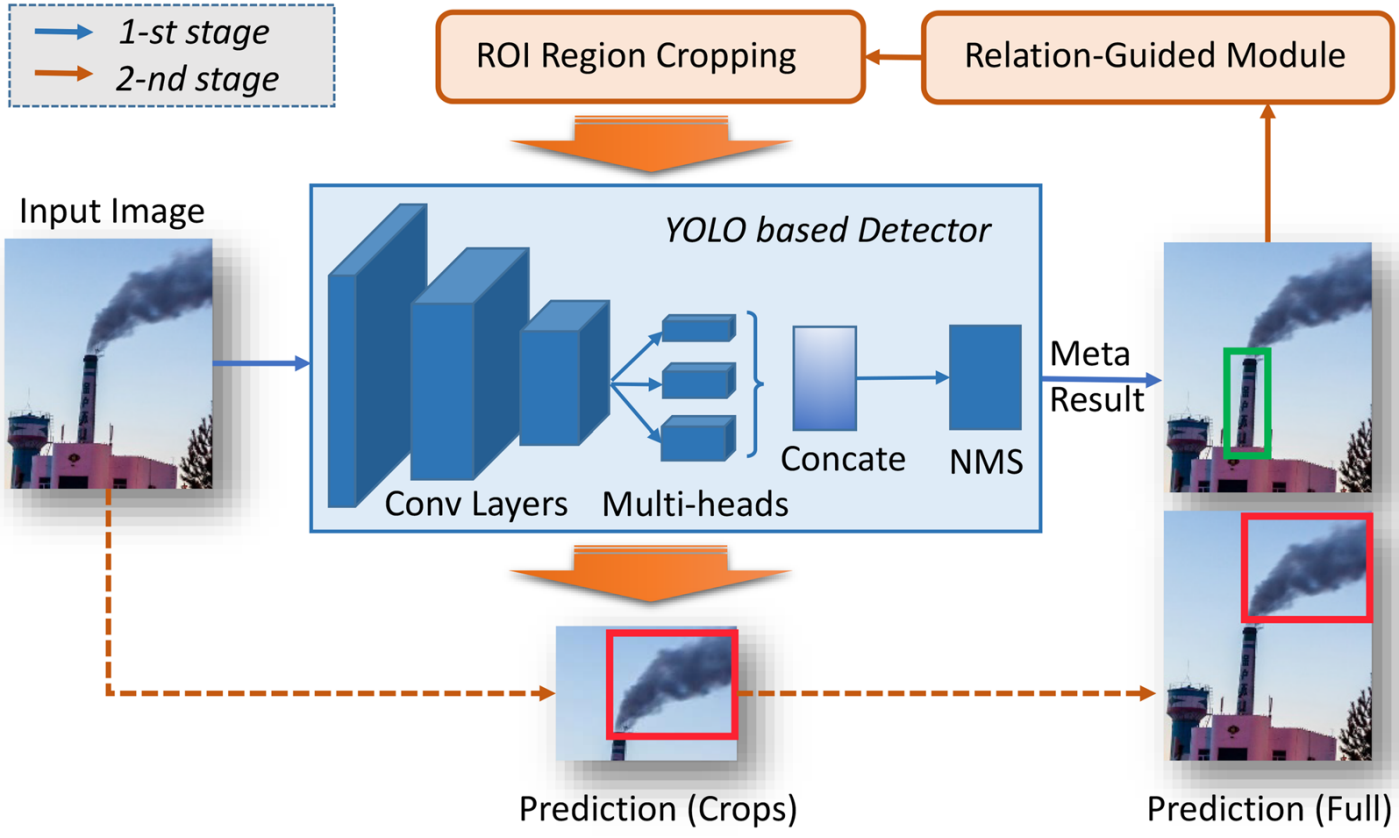
The framework of TSSD algorithm. In the first stage, a YOLO based detector locates chimney in the image, which may produce the meta result as none, one or more prediction boxes; in the second stage, the relation-guided module is to analyze the previous result and carry out ROI region cropping. Then the detector predicts the location of the smoke in the crops, maps this location back to the original image, and eventually outputs the prediction bounding box of factory smoke on the full image.

The framework of TSSD algorithm is shown in Fig. 3. It’s of great importance to design an effective relation-guided module in TSSD algorithm. This module is a bridge to connect two-stage detection, reducing the smoke searing range in the second stage.

### Relation-guided module

The shape and texture of smoke are various and it’s easily affected by weather conditions such as wind, rain and fog, while factory chimney is basically cylindrical and its shape is stable. Therefore, we decide firstly to detect the object easy to detect (chimney) and then to detect the one hard to detect (smoke). What’s more, the smoke is usually located above the factory chimney. This inherent prior knowledge and contextual relation provides the theoretical basis of designing relation-guided module.

We denote the input image height as *h*, width as *w*, and prediction box generated from first-stage detector as $[(x_{min},y_{min}),$ $(x_{max},y_{max})]$, where $(x_{min},y_{min})$ is the upper left coordinate and $(x_{max},y_{max})$ is the lower right one. Due to the influence of the wind direction or shooting angles, the partial smoke may be below the top of the chimney so the smoke shouldn’t be directly detected on $y_{min}$. Supposing that the increased value is $\delta$ times of the length of this box, we obtain a relation value $y_{relation}$ of the height component:

3 $$\begin{aligned} {{y}_{relation}} = {{y}_{min}}+\delta ({{y}_{max}}-{{y}_{min}}) \end{aligned}$$

According to the location relation in our dataset, this paper sets $\delta$ as 0.4 to meet the basic design requirements of the relation-guided module. Using the relation value $y_{relation}$ and the global ROI of the original image, we can get the bounding box $[(0,0),(w,y_{relation})]$ and treats it as the ROI region for the second-stage detection. The range of smoke detection has transformed from the global ROI to local ROI region. It’s exactly the theoretical core of the designed relation-guided module. In this way, searching space of the detector is reduced, which is conducive to finer regression of neural network.

**Figure 4:**
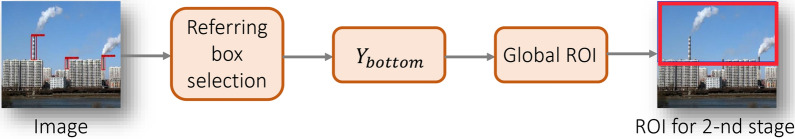
The function diagram of multi-object relation-guided module. For three prediction boxes from the first stage, this module selects $Y_{bottom}$ of the biggest *Y* value at the top left vertex and finds the corresponding box. In combination with the global ROI, it further determines the detected ROI region in the second stage. Please refer to “”Relation-guided module” for the process of the detailed calculation.

However, the above calculation is only suitable to the single prediction box from the first-stage detector. When multiple prediction boxes are generated, this paper uses the multi-object relation-guided module to solve this task. The function diagram of this module is shown in Fig. 4. For the prediction boxes in the figure, we represent their coordinates as $[(x_{i,min},y_{i,min}),(x_{i,max},y_{i,max})]$ (i = 1,2.3), from left to right. If to apply the leftmost prediction box to obtain the new detection region based on Eq. (3), the part of the rightmost smoke will miss detection. This paper introduces the function max{} to obtain the maximum $y_{min}$ value as $Y_{bottom}$ to handle.

4 $$\begin{aligned} Y_{bottom}=\max \{{{y}_{1,min}},{{y}_{2,min}},{{y}_{3,min}}\} \end{aligned}$$

Supposing that $$Y_{bottom}$$ = $$y_{2,min}$$, the relation-guided module can select the prediction box $$[(x_{2,min},y_{2,min} ),(x_{2,max},$$ $$y_{2,max})]$$ to obtain $$y_{relation}$$ based on Eq. (3). Later, the ROI region of the second-stage detection can be obtained by the same way as the relation-guided process for single prediction box. Meanwhile, although the chimney is easy to detect, it may fail to be detected in very few cases. For this issue, this paper directly takes $$y_{relation}$$ as *h*. Then the relation-guided module outputs the bounding box [(0, 0), (*w*, *h*)]. In other words, the original-size image is directly input into the baseline network.

## Experiments

In this section, the image dataset for evaluation is introduced first and then the evaluation metrics are described. Extensive comparsion experiments between TSSD algorithm and other methods are carried out in succession. In addition, we give the intuitive discussion about the visual detection effect of this algorithm.

### Dataset

**Figure 5:**
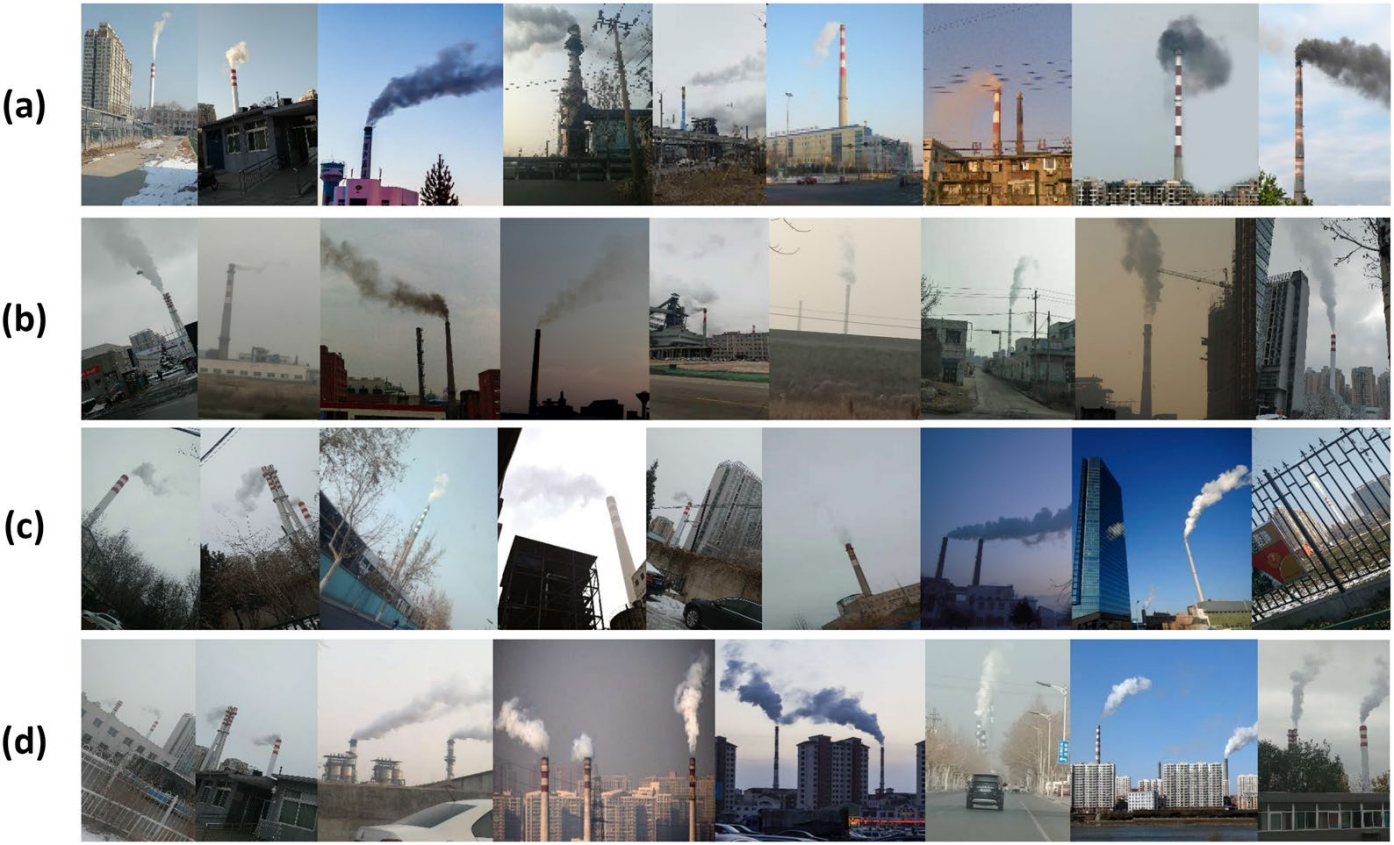
Samples of factory smoke dataset. According to image content, they are divided into four classes of **(a–d)**. Class (**a**) refers to smoke images in sunny environment. Class (**b**) corresponds to the cloudy environment. The chimney is inclined in class (**c**). There are multiple chimneys and smoke zones in class (**d**).

In order to verify the effectiveness of the proposed TSSD algorithm, we collect a special dataset of 960 factory smoke images, including 500 captured by mobile phones and 460 downloaded from the Internet. The locations of taking pictures are in different cities, such as Beijing and Zibo, in China. All images contain the chimney, the smoke and the background. These similar things also appear in the Internet pictures. The collected dataset can be divided into four classes according to the image content: sunny environment, cloudy environment, smoke tilting, and multiple chimneys. The examples of the factory smoke dataset are shown in Fig. 5.

The transformation is performed by using Python’s *imgaug* toolkit. This toolkit can be downloaded from https://github.com /aleju/imgaug. The transformation details for each image are listed below:

(1) Rotate: take the center point of the picture and rotate it. The angle range is from -30 degrees to 30 degrees. According to the affine transformation, each pixel is rotated to the specified position according to the angle; (2) Flip: flip horizontally, mirror, and swap pixels at corresponding positions; (3) Brightness transform: multiply the value of each pixel by the same number, ranging from 0.5 to 1.5. If it is less than 1, it will become dark; otherwise, it will become bright.

**Figure 6:**
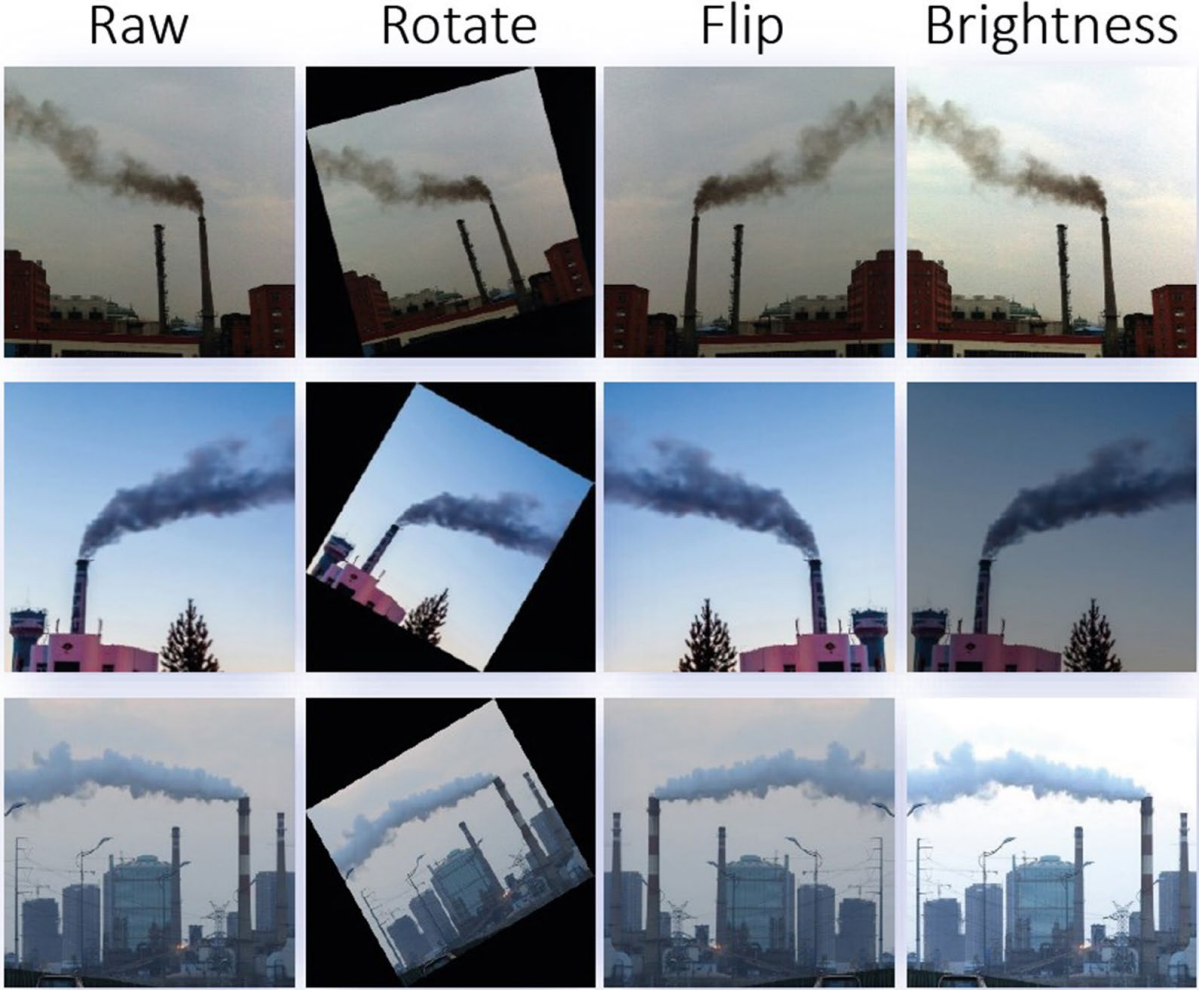
Examples of data augmentation. It includes three kinds of augmentation strategies. The first column is the raw data. The second column is to rotate the image, the third is to flip, and the fourth is to transform darkness.

This dataset is divided according to the training/testing ratio of 7:3, where 672 images act as the training set and the remaining ones as the test. To avoid the over-fitting problem from the few-shot training and learn more essential smoke features by neural networks, we adopt data augmentation strategies to expand the training set and enlarge the diversity of samples, including small-angle rotation, horizontal flip and brightness transform. Using these methods, one factory smoke image can augment to three ones. The details of the dataset distribution are shown in Table 1. The effect of data augmentation is shown in Fig. 6.

**Table 1:** The detailed distribution of the dataset.

Source	Mobile phones	Internet	Original	Augmentation	Whole set
Train set	370	302	672	2016	2688
Test set	130	158	288	/	288
Total	500	460	960	2016	2976

### Evaluation metrics

In this paper, the performances of TSSD algorithm and compared methods are evaluated by the following metrics:

#### Average precision (AP)

We denote the precision rate as P and the recall rate as R. In general, the increase of the precision rate is synchronous with the decrease of the recall rate. To balance them better, PR curve is used to describe the performance of TSSD algorithm. The area under the curve is AP value. Because it’s necessary to locate factory smoke with high accuracy in this research, the AP@IoU$=$0.65:0.05:0.8 is taken as the reference metric. That is marked as AP@65$\sim$AP@80.

#### Mean of different AP values ($AP_{mean}$)

To fairly compare TSSD algorithm with mainstream object detection methods, we refer to the evaluation metric^36^ on the COCO dataset^37^ and mark the mean of AP@50$\sim$AP@95 as $AP_{mean}$. It should be noted that this task involves only smoke class.

#### Inference speed

Model speed is measured by the inference time and FPS (Frames Per Second). The inference time represents the forward time of the neural network detecting one smoke image, while FPS shows the number of the network detecting images per second.

### Experimental details

The experimental environment of TSSD algorithm is: in terms of hardware, we adopt the Intel (R) Xeon (R) CPU e5-2660 processor and the graphics card of GeForce RTX 2080 Ti. In terms of software, we choose the Ubuntu 16.04 operating system, TensorFlow1.12.0 deep learning framework, and Python3.6 programming language.

#### Training details

TSSD algorithm uses the original images and chimney labels as input into the baseline network to train first, which can get a detection model of the chimney. Then, the designed relation-guided module analyses and processes chimney labels to output the reduced smoke detection range. The images of this region and smoke labels are input into the baseline network for training, which can get a smoke detection model. General settings are as follows. The size of network input images is resized to $416\times 416$. We use a weight decay of 0.0005 and momentum of 0.9, with the batch size of 6 and total epochs of 60. To realize better training effect, we set the initial learning rate as 1e−4 and the termination value as 1e−6, and adopt the warmup strategy to adjust it according to the division of the first 30 epochs and the later epochs.

#### Testing details

The testing of TSSD algorithm consists of two stages. In the first stage, we use the trained model of chimney detection to output their prediction boxes. In the second stage, we use the designed relation-guided module to carry out the ROI region cropping for the predicted boxes from the previous stage. Then the trained model of smoke predicts the locations of the smoke in this reduced region. The general setting in the test are as follows. The score_threshold of eliminating redundant prediction boxes is 0.6. The size of the network input is $416\times 416$ except experiments of image resolutions.

### Ablation study of TSSD algorithm

This section carries out the ablation study of TSSD algorithm to prove its superiority over the baseline model. In the training process, there are three values worthy to explore: the IoU threshold $\varepsilon$ involved in the loss calculation, the balance factor $\alpha$ of positive and negative samples, and the hard negative mining coefficient $\gamma$. In addition, whether the trained TSSD model can improve the performance on different image resolutions $\eta$ is also the discussed problem.

#### The training IoU threshold $\varepsilon$

**Figure 7:**
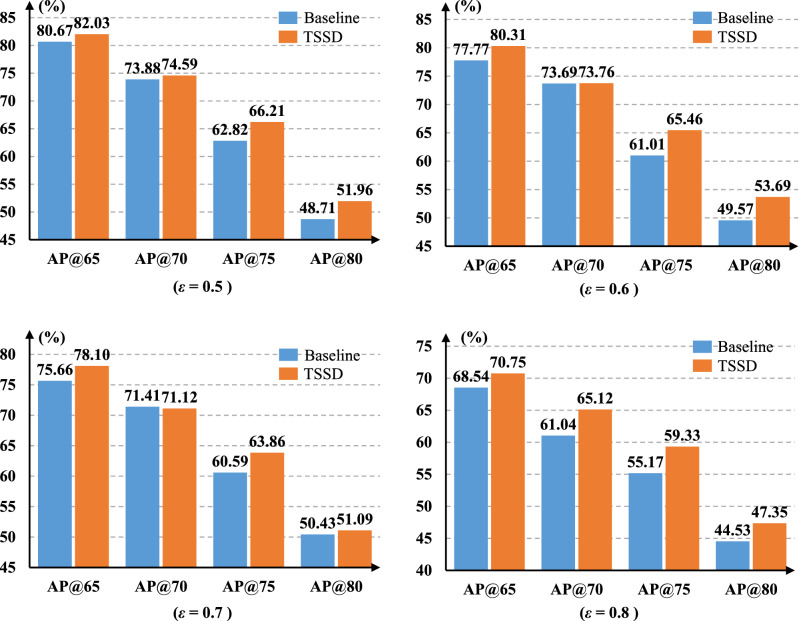
Comparison of the accuracy between TSSD algorithm and the baseline model at different $\varepsilon$ values.

In order to study the individual effect of the IoU threshold $\varepsilon$ on TSSD algorithm, we refer to the best performance of focal loss^34^ on the COCO dataset and fix the parameter $\alpha$ as 0.25 and $\gamma$ as 2. In general, the minimum value of $\varepsilon$ is set as 0.5 to avoid the big scale of detection boxes involving the loss calculation. What’s more, this paper sets the range of $\varepsilon$ as 0.5$\sim$0.8, where the step size is 0.1. This aims to compare TSSD with the baseline more fully. The experimental results are shown in Fig. 7.

#### The training balance factor $\alpha$

**Figure 8:**
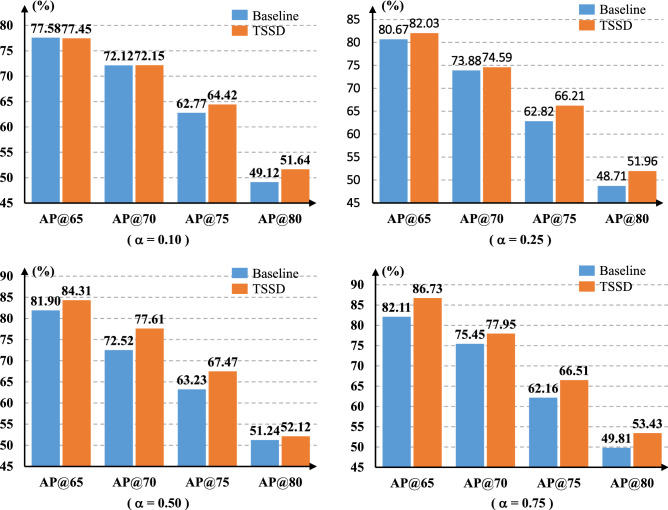
Comparison of accuracy between TSSD algorithm and the baseline model at different $\alpha$ values.

During the training of the baseline network, the proportion of positive and negative samples is 1:10,646. Therefore, it’s of great significance to introduce the balance factor $\alpha$ to relieve the loss gap produced by unbalanced two kinds of samples. To explore the performance of TSSD algorithm with different $\alpha$ values, we fix $\varepsilon$ as 0.5 and $\gamma$ as 2. Referring to the setting of $\alpha$ in focal loss^34^, we take it as {0.10,0.25,0.50,0.75}. Fig. 8 shows the experimental results.

#### The training coefficient $\gamma$

**Figure 9:**
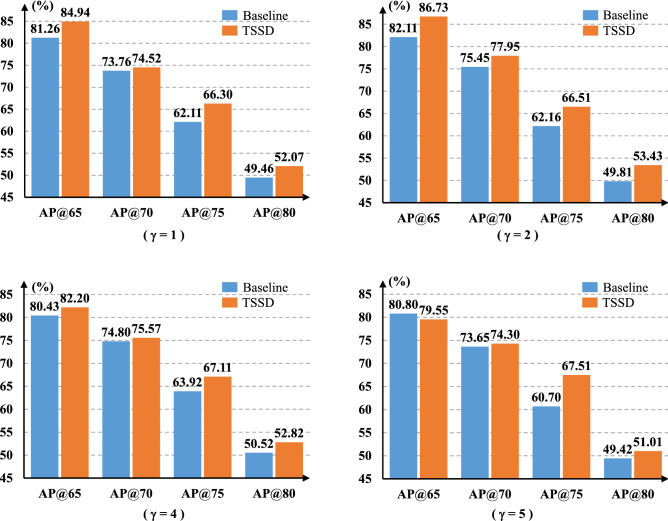
Comparison of accuracy between TSSD algorithm and the baseline model at different $\gamma$ values.

Introducing the coefficient $\gamma$ into the training can reduce the loss influence of simple samples, which enables the neural network to pay more attention to the difficult samples. To verify the performance of TSSD algorithm with different $\gamma$ values, we fix $\varepsilon$ as 0.50 and $\alpha$ as 0.75. In the same way, we refer to the focal loss^34^ and set $\gamma$ as {1,2,4,5}. The experimental results are shown in Fig. 9.

#### The inference image resolution $\eta$

**Figure 10:**
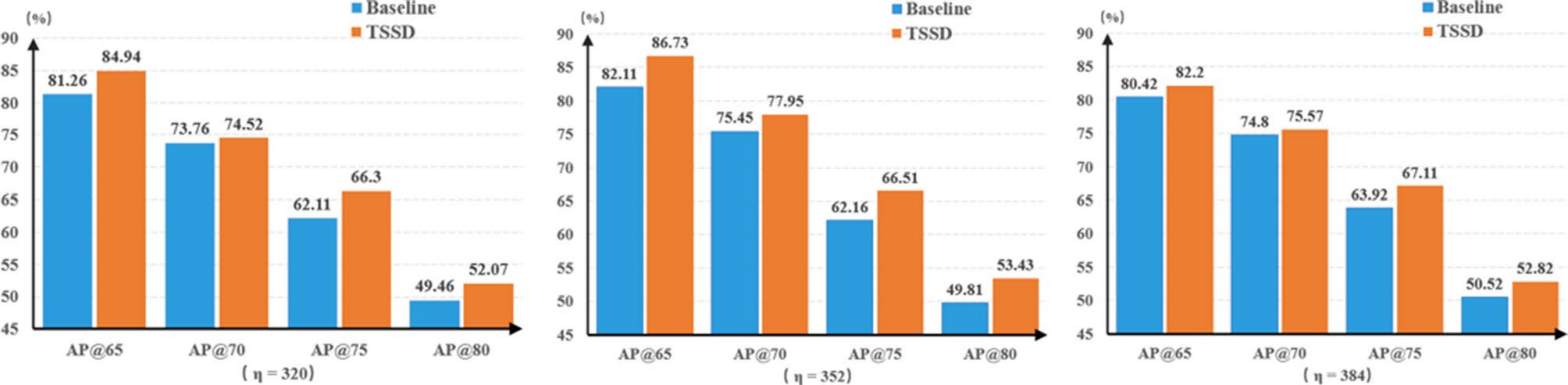
Comparison of accuracy between TSSD algorithm and the baseline model at different $\eta$ values.

Having a good compatibility for different $\eta$ inputs is meaningful for TSSD algorithm. Based on the training parameter set of {$\varepsilon$=0.5,$\alpha$=0.75,$\gamma$=2}, we respectively test the $\eta$ of $320\times 320$, $352\times 352$, and $384\times 384$. The experimental results are shown in Fig. 10.

#### Discussions

From Figs. 7, 8 and 9, it can be clearly seen that TSSD algorithm can steadily improve the detection accuracy of the baseline when to change one of {$\varepsilon$, $\alpha$, $\gamma$} parameters. The conclusion is still valid for different $\eta$ sets based on Figure 10. This strongly proves the effectiveness of TSSD algorithm. Especially, from Fig. 7, when to set different $\varepsilon$ values, AP@65 and AP@80 of TSSD algorithm can obtain the improvement of over 2$\%$ and AP@75 over 3$\%$ than the baseline model. In addition, the biggest gain of 4.45$\%$ can be got when $\varepsilon$ is 0.6 with AP@75. According to Fig. 8, when to set $\alpha$ as 0.50 or 0.75, AP@65$\sim$AP@75 of TSSD algorithm can increase over 3$\%$. Moreover, the highest increase of 5.09$\%$ appears at $\alpha$ as 0.50 with AP@70. On the basis of Fig. 9, for all the mentioned $\gamma$ parameter settings, AP@75 of TSSD algorithm can realize the raise of over 3 $\%$ and AP@80 over 2$\%$. Meanwhile, the best raise occurs at $\gamma$ as 5 with AP@75. In Table 2, when $\eta$ is $320\times 320$ or $384\times 384$, AP@75 and AP@80 of TSSD algorithm can increase over 3$\%$, and AP@65$\sim$AP@80 gets the gain of over 2$\%$ at $\eta$ as $352\times 352$.

TSSD algorithm realizes so outstanding performance on different training parameters and we think there are three reasons as follows. (1) The relation-guided module in TSSD algorithm can transform the range of detection from the global ROI to the local ROI, which undoubtedly reduces the searching space of the needed object. This helps the neural network achieve more accurate regression for the object’s bounding boxes, determining the finer location of the object. (2) Prior knowledge information is introduced into TSSD algorithm, so that the reduced-range images must contain the smoke object, which improves the certainty of object detection. (3) The relation-guided module effectively eliminates the interference of the objects similar to smoke outside the reduced region.

In addition, by resizing to change $\eta$, it only has a certain influence on the clarity of images. That doesn’t damage the inherent location relation between the smoke and the chimney. In other words, the optimization strategy of TSSD algorithm stills works and is not affected.

### Comparison with the state-of-the-art detection methods

To verify the comprehensive performance of TSSD algorithm, we compared it with various state-of-the-art detection methods, including Faster RCNN^17^, SSD^18^ and the baseline model. For Faster RCNN, we choose Resnet50 and Resnet101^38^ as the feature extraction networks. For SSD, we use Inception-v2^39^ and MobileNet-v2^40^.

**Figure 11:**
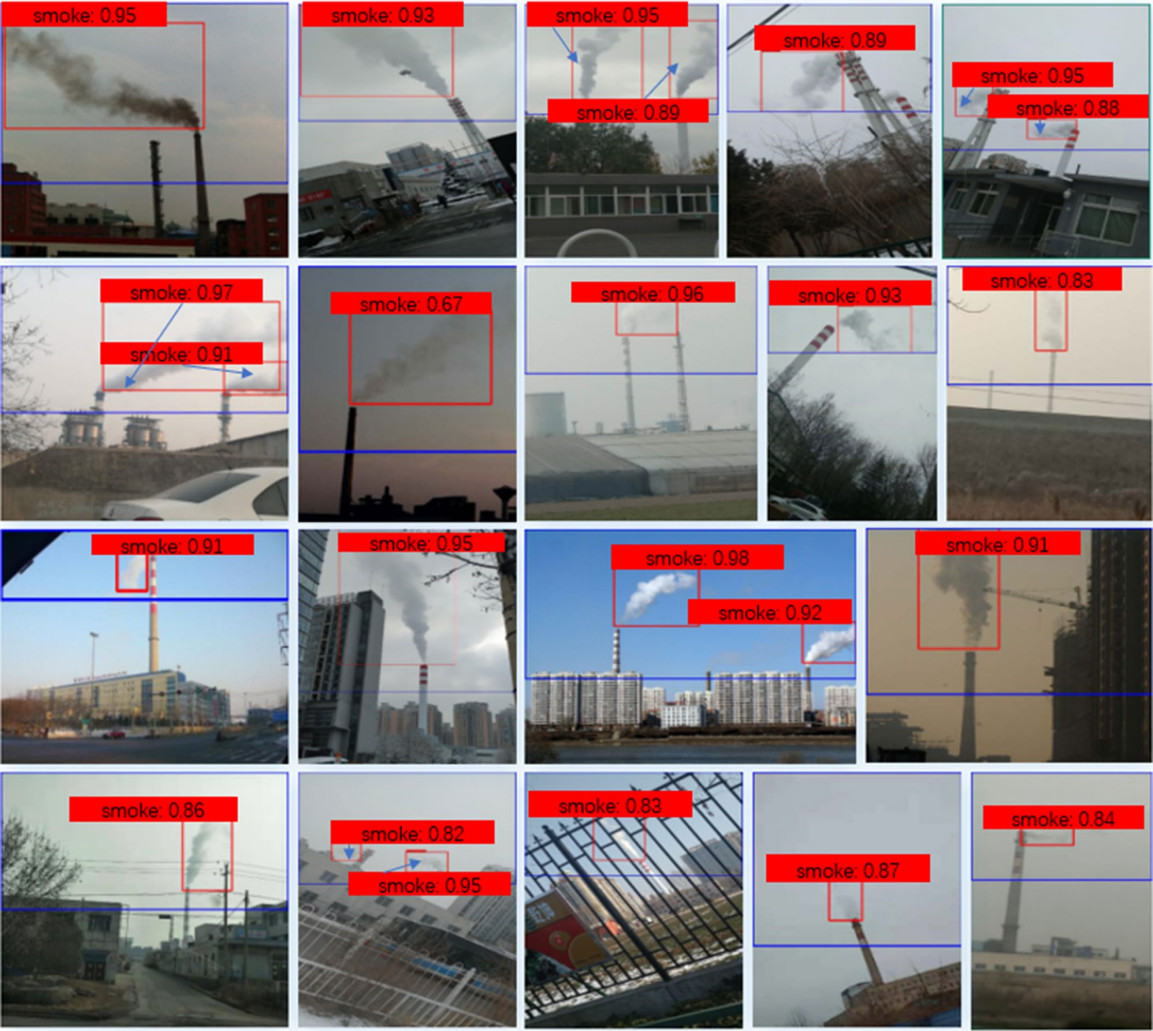
Visual effect of TSSD algorithm on detecting factory smoke.Four-class smoke images of dataset are all included. Blue boxes represent the reduced detection range, and red boxes show the detected smoke region. The text on top of each bounding box represents the confidence of smoke detection.

To fairly compare all the models, the size of the network input is set as $416\times 416$, and their training is based on the pre-trained weight on the COCO. Faster RCNN and SSD are trained until the loss function converges with the stable accuracy. The baseline model and TSSD algorithm adopt the same training parameter settings {$\varepsilon$=0.5,$\alpha$=0.75,$\gamma$=2}. For the fair evaluation between these models, we choose $AP_{mean}$ as the accuracy metric. The experimental results are shown in Table 2, where the inference speed of TSSD algorithm is the time sum of the two stages. The effectiveness of it is intuitively visualized in Fig. 11.

**Table 2:** Comparison of TSSD algorithm and various state-of-the-art detection methods.

Detection methods	Feature networks	$AP_{mean}$ (%)	Time (ms)	FPS
Faster RCNN^17^	Resnet101	58.76	78	13
Faster RCNN^17^	Resnet50	57.82	72	14
SSD^18^	Inception-v2	50.54	25	40
SSD^18^	MobileNet-v2	50.82	**22**	**45**
Baseline	Darknet53	57.16	24	42
TSSD (ours)	Darknet53	**59.24**	50	20

Significant values are in bold.

From Table 2, it’s known that the detection accuracy of TSSD model is 59.24$\%$. It reaches the highest accuracy, even surpassing the current detection model Faster RCNN101. Although TSSD model is slower than the fastest model SSD_Mobilenet-v2, it has the accuracy improvement of 8.42$\%$. Meanwhile, the speed of TSSD model is 50 ms (20 FPS), meeting the need of real-time detection. All of these show that our proposed TSSD algorithm has a bigger advantage than other methods.

We think there are two primary reasons for such superiority. (1) The baseline model of TSSD algorithm is suitable for this task. Its detection accuracy is only 1.6$\%$ lower than Faster RCNN_Resnet101, but the speed is 3.25 times faster. Although the speed is slightly slower than SSD_Mobilenet-V2, its accuracy is 6.34$\%$ higher. (2) The TSSD can robustly improve the accuracy of the baseline model. The specific reasons can be seen in “Ablation Study of TSSD algorithm”.

## Conclusion

This paper proposes a two-stage relation-guided smoke detection (TSSD) algorithm, which can be used on the mobile platform. This algorithm realizes the real-time and high-accuracy smoke location. Compared with the baseline model, TSSD algorithm can robustly improve the detection accuracy under different training parameters, and it also has a good adaptability to different image resolution inputs. Compared with state-of-the-art detection methods, TSSD algorithm achieves the highest accuracy(59.24%), and its speed (20 FPS) can meet the real-time requirements. These embody the effectiveness of the proposed TSSD algorithm in this task. It provides the environmental protection department with an auxiliary means of monitoring factory smoke pollution, having the practical value and a great social supervision significance.

As mentioned above, this paper is mainly aimed at the location and recognition of the factory smoke. It’s still a little difficult to classify and identify whether the detected smoke is harmful. In the future work, we will explore to jointly predict the smoke location and the pollution degree.
